# Change in effectiveness of mammography screening with decreasing breast cancer mortality: a population-based study

**DOI:** 10.1093/eurpub/ckac047

**Published:** 2022-06-23

**Authors:** Søren R Christiansen, Philippe Autier, Henrik Støvring

**Affiliations:** Department of Public Health, Aarhus University, 8000 Aarhus C, Denmark; Institute of Global Public Health, University of Strathclyde at the International Prevention Research Institute, Lyon 69570, France; Department of Public Health, Aarhus University, 8000 Aarhus C, Denmark

## Abstract

**Background:**

Reductions in breast cancer mortality observed over the last three decades are partly due to improved patient management, which may erode the benefit-harm balance of mammography screening.

**Methods:**

We estimated the numbers of women needed to invite (NNI) to prevent one breast cancer death within 10 years. Four scenarios of screening effectiveness (5–20% mortality reduction) were applied on 10,580 breast cancer deaths among Norwegian women aged 50–75 years from 1986 to 2016. We used three scenarios of overdiagnosis (10–40% excess breast cancers during screening period) for estimating ratios of numbers of overdiagnosed breast cancers for each breast cancer death prevented.

**Results:**

Under the base case scenario of 20% breast cancer mortality reduction and 20% overdiagnosis, the NNI rose from 731 (95% CI: 644–830) women in 1996 to 1364 (95% CI: 1181–1577) women in 2016, while the number of women with overdiagnosed cancer for each breast cancer death prevented rose from 3.2 in 1996 to 5.4 in 2016. For a mortality reduction of 8.7%, the ratio of overdiagnosed breast cancers per breast cancer death prevented rose from 7.4 in 1996 to 14.0 in 2016. For a mortality reduction of 5%, the ratio rose from 12.8 in 1996 to 25.2 in 2016.

**Conclusions:**

Due to increasingly potent therapeutic modalities, the benefit in terms of reduced breast cancer mortality declines while the harms, including overdiagnosis, are unaffected. Future improvements in breast cancer patient management will further deteriorate the benefit–harm ratio of screening.

## Introduction

The primary objective of cancer screening and treatment is to reduce the risk of cancer death. Screening may reduce this risk by detecting cancer at an early stage in its development when it is asymptomatic and curable. Treatments generally depend on the stage and other characteristics of the cancer and the patient. Consequently, screening will be most effective if it induces ‘down-staging’, i.e. shifting advanced-stage cancers, for which treatments are less efficient or non-existent, to earlier stages for which effective therapies exist.^1^ The randomized trials of mammography screening showed a reduction of ∼20% in breast cancer mortality among invited women aged 50–70.^2^ The largest reductions were observed in trials conducted before 1985 where few therapeutic options existed for breast cancer. Since then, breast cancer treatments including surgery, radiotherapy and chemotherapy have improved substantially. Consequently, stage-specific survival of breast cancer patients has steadily improved.^3–5^

A recurring question is whether the advent of steadily more effective therapeutic modalities has affected the effectiveness of screening mammography.^6–8^ This may be similar to testis cancer, where the high effectiveness of cis-platinum-based therapies, even for metastatic cancer, has rendered screening obsolete.^9^ In addition, the harms due to screening, mainly the false positive screening test results and the overdiagnosis (i.e. the screen-detection of breast cancer that would not evolve into clinical cancer during a woman’s lifetime), are not reduced by treatment improvement.

A recent study in the USA showed an absolute reduction in the risk of breast cancer death associated with screening of 5 per 10 000 women in the 1970s and 3 per 10 000 women in the 2010s.^10^^,^^11^ The difference in screening effectiveness of 2 per 10 000 women between the 1970s and 2010s was due to the introduction of efficient therapies. This may equivalently be expressed by the number needed to invite (NNI) for avoiding one breast cancer death over 10 years which are here 2000 (i.e. 10 000/5) women in the 1970s and 3333 (i.e. 10 000/3) women in the 2010s.^12^

In most high-income countries, 20–50% decreases in breast cancer mortality have been observed since 1990,^13^^,^^14^ and mortality reductions have consistently been greater for women below screening age.^15^ If mortality decreases were due to factors other than screening, then the number of breast cancer death prevented by mammography screening may have decreased over time. In this article, using data from Norway, we examine the number of women that needs to be invited to screening for preventing one breast cancer death, in the setting of marked changes in breast cancer mortality over time. We also examine the balance between effectiveness and harms using varying levels of overdiagnosis in Norway.

## Methods

### Setting

The Norwegian cancer registry provides reliable registration of cancer incidence and mortality since 1960, well before the results of early randomized trials on screening mammography were published.^16^ The Norwegian mammography screening programme was initially piloted in four counties from 1995 to 1996. The remaining counties were gradually enrolled from 1999 until nationwide coverage was achieved in 2005. Women were invited to mammography screening according to birth cohorts, approximately corresponding to the age range 50–69 years with biennial screening interval.^17^ Around 80% of invited women attend screening mammography.

### Design

We estimated NNI each year from 1986 to 2016 based on estimated breast cancer mortality rates (MR) in a screened Norwegian population and breast cancer MR in the same population without screening. The numbers of women needed to invite to prevent one breast cancer death within 10 years were computed using three inputs: (i) observed breast cancer mortality among Norwegian women aged 50–75 years from 1996 to 2016; (ii) four scenarios of breast cancer mortality reductions over 10 years after screening introductions (i.e. screening effectiveness); and (iii) proportions of breast cancer deaths associated with breast cancers diagnosed before or after screening introduction in the different Norwegian counties. Then after, we used three scenarios of overdiagnosis to estimate the change over time in the ratio of overdiagnosis to breast cancer deaths prevented. Note that the assumed mortality reduction associated with breast cancer screening allows calculation of the hypothetical mortality among invited women, had they not been invited. Similarly among uninvited women, the hypothetical mortality had they been invited can be calculated, as described below.

### Data sources and study population

We retrieved individual data on residence at diagnosis, birth year and time and cause of death for all women diagnosed with breast cancer (ICD10: C50) during the period 1953–2016 from the Cancer Registry of Norway. We followed women until death, emigration, or December 2016, whichever came first. We obtained information on the annual size of the source population in each county and each age from Statistics Norway from 1986 to 2016, such that we could calculate annual MR for this period. The influence of screening on the risk of breast cancer death is to be examined among women in screening ages plus a lead time (6 years, say). Hence, only women aged 50–75 at death were included in analyses, totalizing 10 520 women who died of breast cancer in Norway from 1986 to 2016.

### Statistical analysis

For each breast cancer case, we recorded if the date of diagnosis was before the date of screening introduction in the given county to reduce misclassification of screening status. We aggregated population size, number of deaths and number of unscreened cases for each year and county. Adjustment for change in age distribution over time was done using direct age-standardized rates (DASR).

Using Poisson regression, we estimated the observed breast cancer MR for each year up to 2016. Restricted cubic splines with five knots were used to flexibly adjust for non-linear effects of year at death and reduce the standard error of the estimated rate. Using MR from the regression model, we defined NNI as the number of women 50–69 years of age to be invited to screening within a given year y associated with one less breast cancer death during that year.

(1) $$\mathrm{NN}I_{y}\;=\frac{1}{\mathrm{MR}\;\mathrm{without}\;{\mathrm{screening}}_{y}\;-\;\mathrm{MR}\;\mathrm{with}\;{\mathrm{screening}}_{y}}$$

The MR, and thus the NNI, are estimated in 1-year periods based on women aged 50–75. The 1-year periods facilitates comparison with other studies, which may have differing time-perspectives. The NNI expresses the expected effect of introducing screening in a hitherto unscreened population. We estimated observed MR, which after the introduction of screening is a mixture of the MR in a population without screening and the MR in a population with screening:

(2) $$\mathrm{Observed}\;MR_{y}=\;\mathrm{MR}\;\mathrm{without}\;{\mathrm{screening}}_{y}\;\cdot {\left({\mathrm{prop}}_{\mathrm{ns}}\right)}_{y}\;+\;\mathrm{MR}\;\mathrm{with}\;{\mathrm{screening}}_{y}\cdot \left(1-{\left({\mathrm{prop}}_{\mathrm{ns}}\right)}_{y}\right)$$

In this equation, $\mathrm{pro}p_{\mathrm{ns}}$ is the proportion of women who died in a given year and was diagnosed before introduction of screening in their county. We considered in a first scenario that breast screening can reduce breast cancer mortality by 20% in a population of women aged 50–75 years of age invited to screening. This 20% reduction corresponds to the reduction reported by the Independent Review in UK.^2^ If we let $c_{y}$ be the assumed relative effect of screening (a 20% reduction in breast cancer mortality corresponds to $c_{y}=0.80$) in year y, we have the following relation:

(3) $$\mathrm{MR}\;\mathrm{with}\;{\mathrm{screening}}_{y}=\mathrm{MR}\;\mathrm{without}\;{\mathrm{screening}}_{y}\cdot \;c_{y}$$

Substituting this into the previous formula,^2^ we obtain:

(4) $$\mathrm{MR}\;\mathrm{without}\;{\mathrm{screening}}_{y}=-\frac{\mathrm{Observed}\;{\mathrm{MR}}_{y}}{\left({\left({\mathrm{prop}}_{\mathrm{ns}}\right)}_{y}-1\right)\cdot c_{y}-{\left({\mathrm{prop}}_{\mathrm{ns}}\right)}_{y}}$$

Since the effect of the screening programme is not known and may have changed over time, we considered a range of estimates. Three scenarios had constant relative effects of 20% on breast cancer mortality (Scenario I), 8.7% (Scenario II) or 5% (Scenario IV), respectively, and one scenario had a linearly decreasing relative effect from 20% in 1996 to 8.7% in 2016 (Scenario III). Scenario I is based on the findings of the UK Panel,^2^ whereas Scenario II is based on Birnbaum et al.^11^ who estimated that 15% of advanced-stage cancers were moved to early stage (*in situ* and Stage 1) cancers (see below) by screening. Scenario III is based on assuming a change from the effect observed in randomized trials to a more modern scenario with effect exclusively mediated through a stage shift. Scenario IV is based on a recent study of the Norwegian mammography screening programme.^18^ Although the study could not identify an effect of screening mammography on breast cancer death rates in its main analysis, a quantitative bias analysis suggested that a beneficial effect in the order of a 5% reduction might be compatible with the study’s findings. We consider this a lower bound for any non-negligible effect.

The following formula was used to calculate the effect of screening $c_{y}$ in Scenario II. $\mathrm{pro}p_{\mathrm{ear}}$ is the proportion of early-stage cancers, $\mathrm{pro}p_{\mathrm{adv}}$ is the proportion of advanced-stage cancers, $r_{\mathrm{adv}}$ is the 10-year breast cancer-specific MR for patients diagnosed with advanced-stage cancer and $\mathrm{MR}R_{\mathrm{adv}/\mathrm{ear}}$ is the breast cancer-specific MR ratio comparing patients with advanced-stage cancer to patients with early-stage cancer. We estimated $r_{\mathrm{adv}}$ and $\mathrm{MR}R_{\mathrm{adv}/\mathrm{ear}}$ based on the Norwegian data on stage-specific survival from 2000 to 2016.

(5) $$\frac{\left({\mathrm{prop}}_{\mathrm{adv}}\cdot \left(0.85\cdot r_{\mathrm{adv}}+0.15\cdot \frac{r_{\mathrm{adv}}}{{\mathrm{MRR}}_{\mathrm{adv}/\mathrm{ear}}}\right)+{\mathrm{prop}}_{\mathrm{ear}}\cdot \frac{r_{\mathrm{adv}}}{{\mathrm{MRR}}_{\mathrm{adv}/\mathrm{ear}}}\right)}{{\mathrm{prop}}_{\mathrm{adv}}\cdot \left(1\cdot r_{\mathrm{adv}}\right)+{\mathrm{prop}}_{\mathrm{ear}}\cdot \frac{r_{\mathrm{adv}}}{{\mathrm{MRR}}_{\mathrm{adv}/\mathrm{ear}}}}=1-c_{y}$$

The numerator reflects breast cancer-specific mortality when 15% of patients, who would have had a clinical diagnosis with advanced-stage cancer, instead have their cancer detected as early-stage due to screening, whereas the denominator gives the breast cancer-specific mortality without screening, i.e. without a stage-shift. Based on the estimated Norwegian rates and this formula, we estimate a relative effect of screening on breast cancer mortality of 8.7%, i.e. $c_{y}$ = 0.923.

We weighted the MR of each county by the inverse of the square of the SE to estimate a national breast cancer MR for each year (1986–2016). 95% CI for NNI were estimated via the 95% CI for the denominator of the NNI calculation. The SE for the denominator is estimated as follows:

(6) $$\mathrm{SE}\left(\log\left(\mathrm{MR}\;\mathrm{without}\;\mathrm{screenin}g_{y}\;-\;\mathrm{MR}\;\mathrm{with}\;\mathrm{screenin}g_{y}\right)\right)=\sqrt{\left(1+c_{y}^{2}\right)}\cdot \frac{1}{\sqrt{N_{y}}}$$

In this equation, N is the number of observed deaths.

In addition, we repeated the analysis with DASR to take the change in age distribution into account. The standard population was set to the Norwegian female population in 1996 to show how the effect of mammography screening would have changed had the age distribution been held constant since the first introduction of screening in a Norwegian county. We consider the DASR analysis as our primary analysis, as it facilitates comparisons with other countries. The non-standardized analysis shows the development of the actual population in Norway invited to screening (see Supplementary material S1).

Using the same models as above, but substituting incidence rates for MR (Formulas 1 and 4), we could similarly estimate NNI to overdiagnose one woman to compare the benefits of screening with the harms. Again, the starting point was observed rates coupled with an assumed level of overdiagnosis. The Research Council of Norway has estimated that 17.5% of breast cancers found among women invited to screening would represent overdiagnosis,^19^ a figure in line with the 20% reported by UK independent Panel for women based on the Malmo and Canadian trials.^2^ Overdiagnosis is defined as the proportion of excess cancers among cancers diagnosed during screening period in women invited for screening, definition C in the Independent UK Panel.^20^ We took 20% for overdiagnosis Scenario A. In addition, we considered alternative scenarios with 10% (Scenario B) and 40% overdiagnosis (Scenario C), respectively. As a measure of the benefit–harm ratio, we divided the NNI for overdiagnosis by the corresponding NNI for saving a woman from dying of breast cancer within 10 years.

All analyses were performed using STATA^®^ version 15.1.

### IRB approval

The study has used data from the Cancer Registry of Norway.

The interpretation and reporting of these data are the sole responsibility of the authors, and no endorsement by the Cancer Registry of Norway is intended nor should be inferred. No further approval from an ethics committee was needed to conduct the study.

## Results

**Figure 1:**
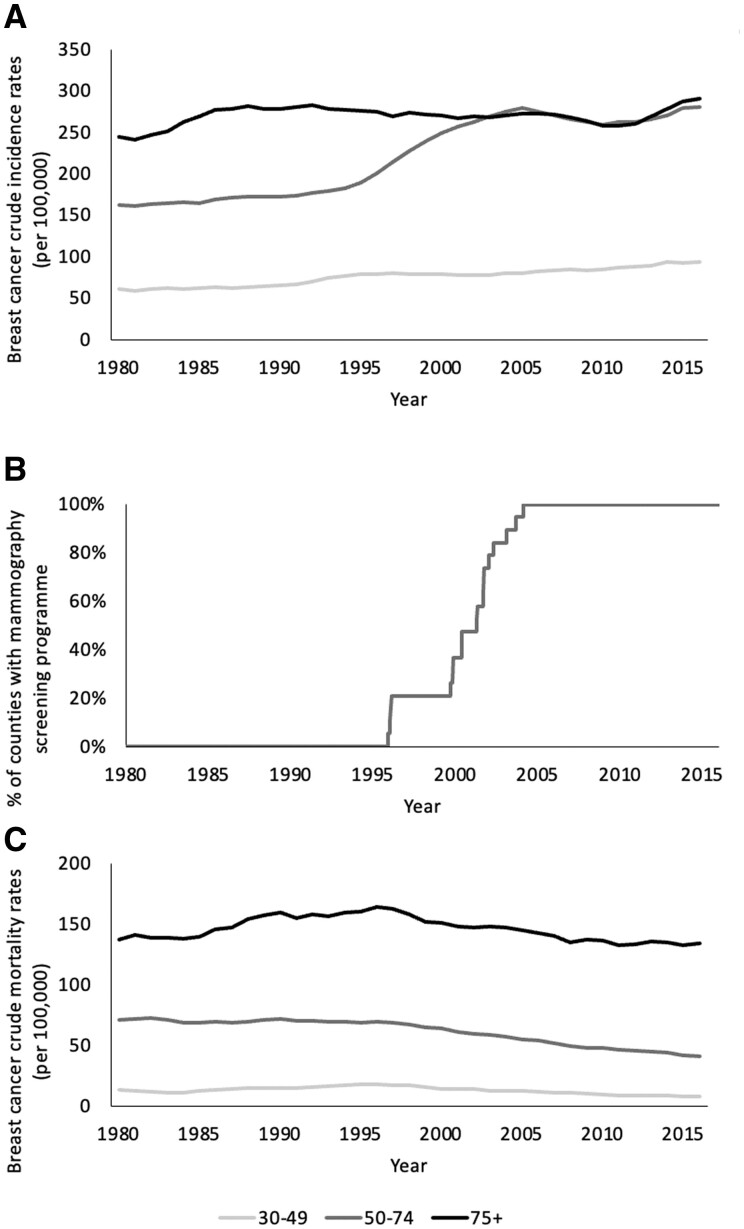
Crude breast cancer incidence (A), proportion of counties with mammography screening programme (B) and crude breast cancer MR (C) among Norwegian women, 1980–2016. Based on publicly available NORDCAN data.^21^ Note that the incidence and MR are 5-year moving average (e.g. the rate in 2000 is the average of 1996–2000).

From 1980 to 1996, incidence rates of invasive breast cancer in Norway steadily increased in all age groups (figure 1A–C). Following breast screening introduction in 1996, sharp increases in incidence were observed among women aged 50–75 years of age, but not in other age groups. After an increase after 1980, breast cancer mortality stabilized around 1985. In 1995, mortality started to markedly decline in all age groups, and from 1996 to 2016, age-adjusted breast cancer death rates dropped by 44% from 81.2 to 49.0 per 100 000 women aged 50–74 years.^21^

During the period 1986–2016, a total of 10 580 breast cancer deaths were identified for women aged 50–75 at death. We excluded 60 deaths because of missing information regarding county. A total of 10 520 deaths were included in the analysis (Supplementary table S1).

The numbers of women who need to be invited to screening associated with one less breast cancer death within 10 years (NNI) in 1996 and 2016 taking a time horizon of 10 years are summarized in table 1 together with ratios of numbers of overdiagnosed breast cancers per breast cancer death prevented thanks to screening.

**Table 1:** Age*-*standardized NNI to avoid one breast cancer death and the number of women with overdiagnosed breast cancer per BC death prevented

**Scenarios of screening effectiveness** ^a^	Year	NNI and 95% CI, 10-year follow-up	Change in NNI (%)	No. women with overdiagnosed BC per BC death prevented
Scenario I (20%)	1996	731 (644–830)		3.2
	2016	1364 (1181–1577)	87	5.4
Scenario II (8.7%)	1996	1685(1474–1926)		7.4
	2016	3518 (3019–4099)	109	14.0
Scenario III (20–8.7%)	1996	731 (644–830)		3.2
	2016	3518 (3019–4099)	381	14.0
Scenario IV (5%)	1996	2934 (2560–3362)		12.8
	2016	6338 (5423–7406)	116	25.2

Notes: Level of overdiagnosis is assumed to be 20% (overdiagnosis Scenario B). The change describes the increase in NNI from 1996 to 2016.

a % reduction in breast cancer mortality associated with screening mammography. See the Methods section.

NNI, number needed to invite, i.e. the number of Norwegian women 50–69 years of age who need to be invited to screening for avoiding one breast cancer death during the subsequent 10-year period; BC, breast cancer.

The observed breast cancer mortality has decreased 46% from 74 per 100 000 in 1986 to 40 per 100 000 in 2016 in women aged 50–75. In Scenario I, screening is assumed to reduce the breast cancer mortality by 20% throughout the observation period. NNI was estimated as 731 (95% CI: 644–830) women in 1996, and 1364 (95% CI: 1181–1577) women in 2016, which corresponds to an increase in NNI by 87%. Scenario II with a constant screening effect of 8.7% estimated the NNI in 1996 as 1685 (95% CI: 1474–1926) women and 3318 (95% CI: 3019–4099) women in 2016 corresponding to a relative increase of NNI by 97%. Scenario III with at linear developing screening effect from 20% in 1996 to 8.7% in 2016 yielded an estimated 354% increase in NNI from 731 (95% CI: 644–830) women in 1996 to 3318 (95% CI: 3019–4099) women in 2016. Scenario IV with a constant screening effect of 5% estimated the NNI in 1996 as 2934 (95% CI: 2560–3362) women and 6338 (95% CI: 5423–7406) women in 2016 corresponding to a relative increase of NNI by 116%. Time changes in NNI are graphically displayed in Supplementary figures S1 and S2.

Because overdiagnosis is related to screening and unrelated to changes in mortality, the number of women with overdiagnosed breast cancer relative to NNI to save one woman from dying of breast cancer nearly doubled from 1996 to 2016 in every scenario with and without age-adjustment (table 2).

**Table 2:** Age*-*standardized NNI to overdiagnose one woman and the number of women with overdiagnosed breast cancer per breast cancer death prevented

**Scenarios of overdiagnosis** ^a^	Year	NNI and 95% CI, 10-year follow-up	Change in NNI (%)	No. women with overdiagnosed BC^b^ per BC death prevented
Scenario A (10%)	1996	439 (406–475)		1.7
	2016	462 (435–491)	5	3.0
Scenario B (20%)	1996	229 (211–248)		3.2
	2016	252 (236–269)	10	5.4
Scenario C (40%)	1996	123 (113–135)		5.9
	2016	147 (137–158)	20	9.3

Notes: Level of screening effectiveness is assumed to be 20% (screening effectiveness Scenario I). The change describes the increase in NNI from 1996 to 2016.

NNI, number needed to invite, i.e. the number of Norwegian women 50–69 years of age who need to be invited to screening for one woman to be overdiagnosed during the subsequent 10-year period;

a % overdiagnosis associated with screening mammography.

b BC, breast cancer.

The joint effects of screening effectiveness and of overdiagnosis result in increasing ratios of overdiagnosed breast cancers per breast cancer death prevented from before 1990 when efficient therapies were not available to years when patient management was progressively more efficient (table 3). In addition, decreasing screening effectiveness leads to marked increases in ratios of overdiagnosed breast cancers for each breast cancer death prevented.

**Table 3:** Ratios of the number of overdiagnosed women per breast cancer death prevented according to scenarios outlined in tables 1 and 2

	Overdiagnosis scenarios					
	10%		20%		40%	
Screening effectiveness scenarios (%)	1996	2016	1996	2016	1996	2016
20	1.7	3.0	3.2	5.4	5.9	9.3
8.7	3.8	7.6	7.4	14.0	13.7	23.9
20–8.7	1.7	7.6	3.2	14.0	5.9	23.9
5	6.7	13.7	12.8	25.2	23.9	43.1

## Discussion

This population-based study found a substantial increase over time in numbers of Norwegian women aged 50–69 who need to be invited to screening mammography in order to prevent one breast cancer death. The increase was a consequence of the substantial decrease in breast cancer mortality among Norwegian women aged 50–75. As overdiagnosis is unaffected by changes in screening effectiveness the ratio of overdiagnosed breast cancers for each breast cancer death increased markedly over time.

Our results follow the logic that if deaths due to a cancer become rarer, the absolute mortality risk reduction due to screening for that cancer will be diminished. Consequently, the number of subjects who need to be invited for preventing one breast cancer death will increase with the availability of increasingly potent therapeutic modalities. In addition, the NNI will be further inflated if the ability of screening to reduce the risk of cancer death proves lower than the 20% reduction suggested by randomized trials.^2^^,^^22^ The continued decrease in mortality in Norway after the mid-1990s (figure 1A and B) could only be a screening effect if the relative effect of screening had increased over the period, which seems implausible. The declining mortality, also seen in unscreened age groups, is therefore also an effect of improved patient management.

A major strength of this study is that it is based on the nationwide Norwegian population invited to mammography screening, which virtually eliminates selection bias. The year-by-year estimation of NNI has allowed more accurate estimation of changes in NNI due to factors other than screening effectiveness.

A potential limitation is that some women who died at the earliest ages of the screening age will not have had an effect of screening, since they will have been diagnosed before receiving the invitation to the screening programme. This misclassification is however conservative as it would lead to an underestimation of NNI throughout the period.

Previous studies that estimated NNI for screening mammography obtained highly variable results depending on the absolute reduction in breast cancer MR considered, the age range of screened women, the time horizon, and whether the NNI was estimated for women invited to screening or for women who were actually screened (reviewed in 23) In any event, NNI estimates in previous studies (including those of the UK Panel) are not contemporary as they are based on historical data before more widespread use of tamoxifen and do not account for any change in breast cancer mortality reductions due to improved patient management. In principle, improved management may increase the number of women who could benefit from earlier detection through screening, which is a limitation to our results. However, a previous study has found that relative screening benefits would be unaffected by improved management.^11^

Because of the unknown effect of screening on breast cancer mortality in the Norwegian female population, we had recourse to scenarios based on plausible estimates for screening effectiveness. Moreover, the way we computed NNI allows comparisons between studies. The UK Panel found an NNI of 235 women invited during 20 years based on UK data with a screening effect based on a review of randomized trials from 1963 to 1991 (i.e. 20% reduction in breast cancer mortality and 20% overdiagnosis associated with screening).^2^ Our analyses found NNI values for 2009 somewhat higher (379 vs. 235; Supplementary table S2), likely due to considering a younger age interval for breast cancer mortality.^20^ Regardless, the most important result is the 87% NNI increase with age-adjustment from first introduction of mammography screening in 1996 until 2016 (table 1). Similarly, the level of overdiagnosis remains a contentious issue. We therefore included three different scenarios with 10%, 20% and 40% overdiagnosis, which all showed more than a 50% increase in the number of women overdiagnosed per breast cancer death prevented from 1996 to 2016 after age standardization with 20% breast cancer mortality reduction.

Autier et al.^24^ were the first to investigate the development in NNI as a result of an assumed decrease in breast cancer mortality. They estimated an increase in NNI of 50% from 952 women invited during 10 years in 2001 to 1429 women invited during 10 years in 2015 based on an assumed 50% decrease in breast cancer mortality from 1985 to 2015 unrelated to screening and a screening effect of 20% (Supplementary table S2). If we were to assume a similar screening effect, we estimate a 41% increase in NNI over the same period without age-adjustment. However, this study is not based on assumed decreases in breast cancer mortality, but on decreases observed in Norway. Likewise, the Scenario II is based on the method used by Birnbaum et al.,^11^ but applied to Norwegian data instead of US SEER data. Owing to differences in mortality trends over time and in age-specific MR, the NNI found in Scenario II are comparable to NNI found using US SEER data. More importantly, increases in NNI with decreasing breast cancer mortality were quite similar in our study and in studies of Birnbaum et al.^11^ and Autier et al.^24^

The adoption of newer screening modalities such as digital mammography or tomosynthesis may have increased screening sensitivity and thus increase screening benefits. However, the Norwegian breast screening programme has demonstrated the absence of added value of tomosynthesis.^25^ Digital mammography is somewhat more sensitive than film-based mammography for very dense breasts but leads to more recalls and more detection of *in situ* breast cancers, without reducing rates of interval cancers.^26^^,^^27^ So, digital mammography may have increased harms associated with screening without meaningful increase in benefits.

In conclusion, what does not change with declining mortality and increasingly potent therapies are the harms due to screening, mainly the overdiagnosis and associated overtreatment. Hence, the benefit-to-harm balance of screening mammography can be expected to increasingly tilt towards the harms, and we expect this deterioration in benefit-to-harm ratio to continue due to ongoing improvement in therapies. A direct consequence of decreasing effectiveness but constant harm is the deterioration of the cost-effectiveness of screening mammography,^28–30^ and our study makes further calls for an analysis of the development in the health and economic costs per woman saved from dying of breast cancer.^31^

## Supplementary data

Supplementary data are available at *EURPUB* online.

## Data availability

The study has used data from the Cancer Registry of Norway. The data included 10 580 women aged 50–69 years diagnosed with breast cancer who died from the disease when aged 50–75 years in 1986–2016 in Norway. Authors are according to Norwegian laws not allowed to share data but refer readers to contact Cancer Registry of Norway to apply for data access.

## Funding

This study is funded by Department of Public Health at Aarhus University (Denmark) and the International Prevention Research Institute, Lyon (France).

*Conflicts of interest*: None declared.

Key points

- Assuming a relative effect of mammography screening at 20% on breast cancer mortality, the number of women who needs to be invited to save one life has increased by 87% from 1996 to 2016.
- The number of women overdiagnosed with breast cancer per woman saved from dying of breast cancer has increased substantially from 1996 to 2016.
- The deterioration in benefit-to-harm ratio of breast screening will continue due to steady improvement in therapies.
- This study supports the need for re-evaluation of national screening programmes in high-income countries.

## Supplementary Material

ckac047_Supplementary_Data
