# Supplementary material for: Change in effectiveness of mammography screening with decreasing breast cancer mortality: a population-based study
Source: Eur J Public Health. 2022 Jun 23;32(4):630–5. doi: 10.1093/eurpub/ckac047 (PMC9341840; doi:10.1093/eurpub/ckac047)
Supplement: ckac047_Supplementary_Data [file ckac047_supplementary_data.zip › ejph-2021-07-om-0850-File002.docx]

Change in effectiveness of mammography screening with decreasing breast cancer mortality: a population-based study

Søren R. Christiansen, Philippe Autier, Henrik Støvring

Supplementary materials

Table S1: Population size and number of breast cancer deaths in Norway

Presented in 5-year age groups and 5-year calendar year groups, unless for the last period 2011-2016 and age group 70-75 which includes 6 years.

|  | 1986-1990 | | 1991-1995 | | 1996-2000 | | 2001-2005 | | 2006-2010 | | 2011-2016 | |
| --- | --- | --- | --- | --- | --- | --- | --- | --- | --- | --- | --- | --- |
| Age | Deaths | PYR^a^ | Deaths | PYR | Deaths | PYR | Deaths | PYR | Deaths | PYR | Deaths | PYR |
| 50-54  55-59  60-64  65-69  70-75 | 211 | 472,977 | 230 | 534,095 | 318 | 705,576 | 282 | 736,514 | 229 | 765,608 | 270 | 965,269 |
|  | 309 | 498,482 | 254 | 464,226 | 311 | 525,650 | 369 | 692,785 | 281 | 725,914 | 317 | 915,846 |
|  | 383 | 546,792 | 314 | 483,094 | 264 | 452,345 | 298 | 510,858 | 358 | 674,432 | 355 | 855,680 |
|  | 454 | 573,257 | 445 | 519,987 | 363 | 461,657 | 280 | 432,618 | 325 | 490,378 | 405 | 789,804 |
|  | 603 | 588,132 | 591 | 620,500 | 543 | 580,849 | 429 | 514,459 | 353 | 484,136 | 436 | 668,792 |

^a^Person years at risk

Table S2: Comparison of NNI in this study to Autier et al. (1) and the Lancet review (2).

Note that the screening effect is assumed to be 20% in all estimates and the age groups vary to make the comparisons possible.

| Year | This study  Women aged 50-75, 10-year follow-up | This study Women aged 50-60, 10-year follow-up ^a^ | Autier et al. Women aged 50-60, 10-year follow-up | This study Women aged 55-75, 20-year follow-up ^b^ | The Lancet Women aged 55-75, 20-year follow-up ^c^ |
| --- | --- | --- | --- | --- | --- |
| 1986 | 683 | 852 | 714 (1985) | 295 | 235 |
| 2001 | 774 | 947 | 952 (2000) | 319 | - |
| 2016 | 1033 | 1.345 | 1.429 (2015) | 435 | - |

^a^The NNI for women aged 50-60 was in this study 8,520 in 1986. This is divided by the 10 years of follow up to compare it with the estimates by Autier et al (1).
^b^The NNI for women aged 55-79 was in this study 5,896 in 1986. This is divided by the 20 years of follow up to compare it with the estimates in the Lancet review (2).
^c^The 20% reduction is based on studies from 1963-91. The mortality rates are based on UK data from an unspecified period.

*Figure S1*: NNI each year associated with one less breast cancer death during that year.

NNI estimated using mortality rates not standardized for age. The lines show the development of NNI in the four scenarios. Scenario I: 20% reduction in breast cancer mortality, scenario II: 8.7% reduction in breast cancer mortality, scenario III: 20% to 8.7% reduction in breast cancer mortality, scenario IV: 5% reduction in breast cancer mortality.

Figure S2: NNI (using direct age-standardized mortality rates) each year associated with one less breast cancer death during that year.

Scenario I: 20% reduction in breast cancer mortality, scenario II: 8.7% reduction in breast cancer mortality, scenario III: 20% to 8.7% reduction in breast cancer mortality, scenario IV: 5% reduction in breast cancer mortality

References

1. Autier P, Boniol M. Mammography screening: A major issue in medicine. European journal of cancer (Oxford, England : 1990). 2018;90:34-62.

2. The independent UK panel on breast ancer screening. The Benefits and Harms of Breast Cancer Screening: An Independent Review. 2012.
